# Playing with Biophysics: How a Symphony of Different Electromagnetic Fields Acts to Reduce the Inflammation in Diabetic Derived Cells

**DOI:** 10.3390/ijms24021754

**Published:** 2023-01-16

**Authors:** Federica Zanotti, Martina Trentini, Ilaria Zanolla, Elena Tiengo, Chiara Mantarro, Luca Dalla Paola, Elena Tremoli, Maria Sambataro, Luisa Sambado, Massimo Picari, Sara Leo, Letizia Ferroni, Barbara Zavan

**Affiliations:** 1 Translational Medicine Department, University of Ferrara, 44121 Ferrara, Italy; 2 Biomedicine Department, University of Ferrara, 44123 Ferrara, Italy; 3 Biomedicine Department, University of Tuscia, 10023 Viterbo, Italy; 4 GVM Care & Research, Maria Cecilia Hospital, 48033 Cotignola, Italy; 5 Endocrine, Metabolism, and Nutrition Disease Unit, Santa Maria di Ca’ Foncello Hospital, 37342 Treviso, Italy

**Keywords:** electromagnetic field, diabetic foot, reactive oxygen species, cytokines, wound healing, inflammation

## Abstract

Several factors, such as ischemia, infection and skin injury impair the wound healing process. One common pathway in all these processes is related to the reactive oxygen species (ROS), whose production plays a vital role in wound healing. In this view, several strategies have been developed to stimulate the activation of the antioxidative system, thereby reducing the damage related to oxidative stress and improving wound healing. For this purpose, complex magnetic fields (CMFs) are used in this work on fibroblast and monocyte cultures derived from diabetic patients in order to evaluate their influence on the ROS production and related wound healing properties. Biocompatibility, cytotoxicity, mitochondrial ROS production and gene expression have been evaluated. The results confirm the complete biocompatibility of the treatment and the lack of side effects on cell physiology following the ISO standard indication. Moreover, the results confirm that the CMF treatment induced a reduction in the ROS production, an increase in the macrophage M2 anti-inflammatory phenotype through the activation of miRNA 5591, a reduction in inflammatory cytokines, such as interleukin-1 (IL-1) and IL-6, an increase in anti-inflammatory ones, such as IL-10 and IL-12 and an increase in the markers related to improved wound healing such as collagen type I and integrins. In conclusion, our findings encourage the use of CMFs for the treatment of diabetic foot.

## 1. Introduction

Diabetic foot, which is the most diffused cause of morbidity among diabetic patients, shows functional and structural alteration such as ulcers often associated with osteomyelitis/gangrene due to chronic inflammation and endothelial dysfunction [1,2]. The endothelial cells, due to the hyperglycemic state, switch from the utilization of nitric oxide to metabolize glucose, the depletion of which results in the inability to vasodilate [3]. The inability to vasodilate increases the intravascular pressure, causing injury and inflammation to the endothelial cells, which in turn causes the subintimal migration of inflammatory cells, thereby inducing the formation of atherogenic foam cells [4]. Moreover, inflammatory cells release lytic enzymes that further damage the extracellular matrix (ECM) and the vessels. This condition induces an imbalance in free radicals and antioxidants, resulting in an overproduction of reactive oxygen species (ROS) [5]. They act as secondary messengers to immune-cells, influence the recruitment and activity of monocytes to the wound site and regulate the angiogenesis process. All these events induce a vasculopathy, which leads to impaired healing and ulceration [6]. In this view, the control of the ROS production represents a promising strategy to improve wound-healing responses, which involves complex processes consisting of distinct, but overlapping phases, such as hemostasis, inflammation, proliferation and remodeling [7]. To this aim, several non-invasive technologies have been developed [8,9,10,11,12,13]. In literature, it is reported that the exposure to a magnetic field is able to favor the anti-inflammatory molecular pathways and can also decrease the production of the ROS [14] in many cellular models. It is well accepted that the devices committed to producing magnetic fields (EMF) are able to affect numerous biological processes, including wound and bone healing, inflammation, osteoarthritis, post-operative edema, chronic/neuropathy pain and tissue repair [9,10,11,12,13,14,15,16,17,18,19,20,21,22,23,24,25]. To ensure safe exposures, exposure guidelines have been regularly developed and revised based on the available scientific knowledge such as the “exposure guidelines” that are published by the International Commission on Non-Ionizing Radiation Protection (ICNIRP). Such guidelines exist for several frequency bands, including low-frequency electric and magnetic fields. The first well accepted and fixed principle is that the exposure to the EMFs in the frequency range of interest do not cause damaging effects to either the patient, the operator or any third party. We worked on EMF for 10 years with different medical devices, studying different models, i.e., skin and bone [26,27,28,29,30,31], confirming their safety and providing both preclinical and clinical data characterized by the coherent data and useful interpretations in order to provide results and be able to replicate them in this fairly “risky” field of research. To this view, this technology can offer an excellent alternative to the classical approaches in all chronic wounds that are as highly invalidating for the patients as Diabetic foot ulcers (DFUs). DFUs are one the most common complications of diabetes mellitus (DM), which often results in disability and is associated with an increased risk of mortality. Studies also confirm that its annual incidence varies from 9.1 million to 26.1 million around the world. DFU pathogenesis generally involves peripheral nerve lesions and peripheral artery diseases. The classical management of DFUs include standard care such as offloading, debridement, moisture-retentive dressings, infection management, tissue-based products, autologous platelet-rich gel and ozone therapy. However, there is limited evidence to support the effectiveness and safety of using EMF as a treatment for DFU, mainly due to the definition of its mode of action and its influence on mitochondrial activity. Due to our previous experience on diabetic cells physiology and on mitochondrial indolence on wound healing [32,33,34,35,36,37,38,39], we focused our attention on testing the effect of a novel system based on complex magnetic fields (CMFs) which consist of a special symphony of waveforms that have been developed for the treatment of several biological alterations. In light of these considerations, the present work aimed to testing the in vitro effects of CMFs on derived diabetics *cells* and evaluating their effect on the mitochondrial ROS production, its related anti-inflammatory activity and its ability to improve wound healing through both molecular (gene expression) and biochemical (ELISA) tests.

## 2. Results

### 2.1. Safety Following Insternational Standard Indication

**Figure 1:**
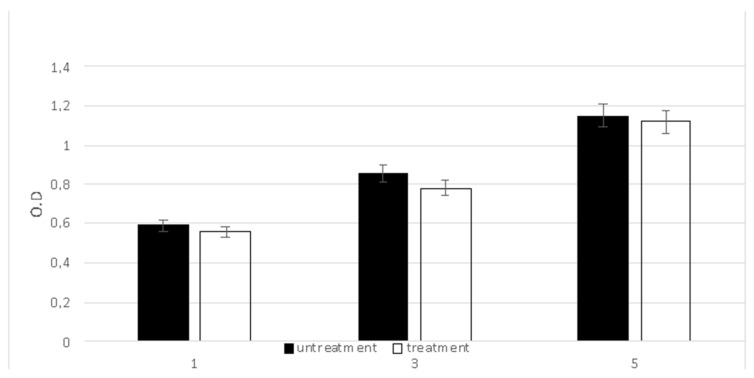
The viability of cells after the CMF treatment with the MTT assay at 1, 3 and 5. Cells cultured in the plastic culture dish were used as control. Data are given as the mean ± standard deviation (n = 3 per group).

When a medical device is required to stay in contact with the human body and when its function influences cellular behavior, excellent biocompatibility is fundamental with the purpose of preventing any adverse effect. In this view, the biocompatibility was evaluated by testing the viability of fibroblastic cells lined with an MTT assay performed after five treatments with a CMF (Figure 1). The cells cultured on the plastic culture dish without treatment were used as a control. The OD values related to the cells that underwent the CMF treatment were very close to those observed in the control sample.

A second test was performed in order to evaluate the blood compatibility by the hemolysis assay that quantifies the free hemoglobin released into the plasma following damage to the blood cells. As reported in Table 1, no hemoglobin was released or detected, confirming the lack of any hemolytic activity due to the CMF treatment.

The mutagenic potential of the CMF treatment was moreover excluded by performing the Ames test. As reported in Table 2, a negative result indicated that the treatment did not induce any mutagenic event.

### 2.2. ROS Production

**Figure 2:**
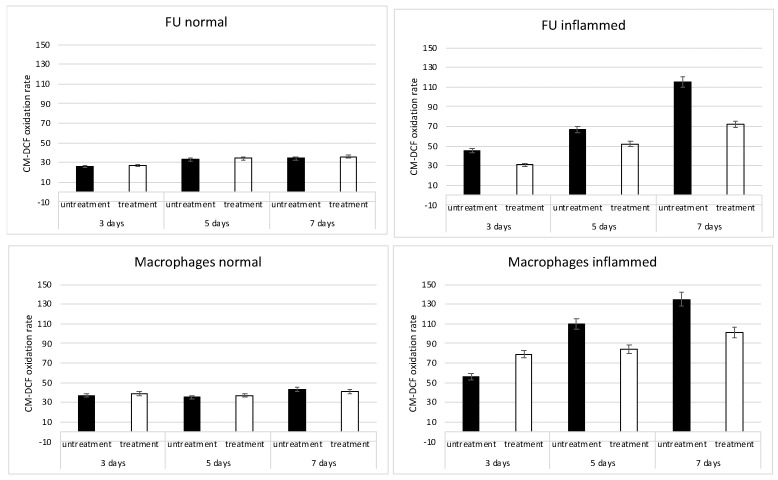
Effects of inflammation on the mitochondrial physiology evaluated by means of the oxidation process activation in fibroblast (FU), inflamed or not, and in macrophages, inflamed or not, in the presence of the CMF treatment (white bars) or in absence (untreated, black bars) with the CMF.

The classical inflammatory environment induced the fibroblasts and macrophages to produce the ROS in a time dependent manner, as reported in Figure 2. The black bars show the absence of any treatment. The treatment of the CMF induced a great reduction in the ROS in both cells type (white bars). This reduction was not present in the normal conditions (left panels), confirming that the CMF acts only in the pathological situation.

### 2.3. Macrophage Polarization

**Figure 3:**
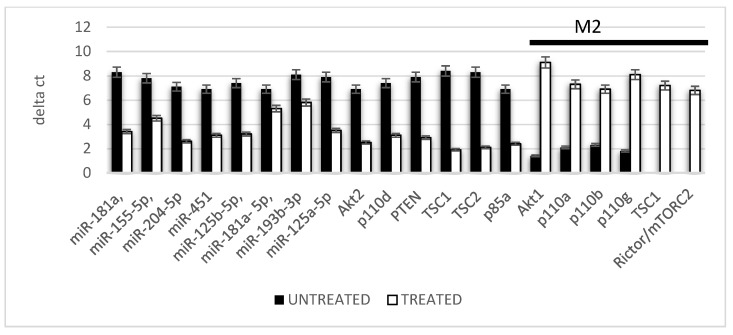
Gene expression related to the macrophages polarization. The genes below the black line are related to the M2 phenotype (M2ϕ). All the macrophages were cultured in the presence of an inflammatory condition; the black bars in absence of the CMF and the white bars in the presence of the CMF.

The polarization of the macrophages on the M1 or M2 phenotype was evaluated by means of the gene expression of genes and miRNAs related to this event. As reported in Figure 3 (black bars), the inflammatory environment usually induced a commitment of the macrophages to an M1 phenotype as reveled by the high expression level of miR-181a, miR-155-5p, miR-204-5p, miR-451, miR-125b-5p, miR-21, miR-193b-3p, miR-125a-5p, Akt2, p110d, PTEN, TSC1, TSC2 and p85a that are gene related to the control of the activation of this commitment pathway. In support of this, it was evident that the gene expression of AKT1, p110a, p110b, p110g, TSC1 and PTEN related to the M2 phenotype was strongly reduced. By contrast, in an inflamed tissue after the CMF treatment, a strong reduction in the expression related to the M1 commitment (white bars) was evident where a strong expression of the genes related to the M2 phenotype (black bar) was increased.

**Figure 4:**
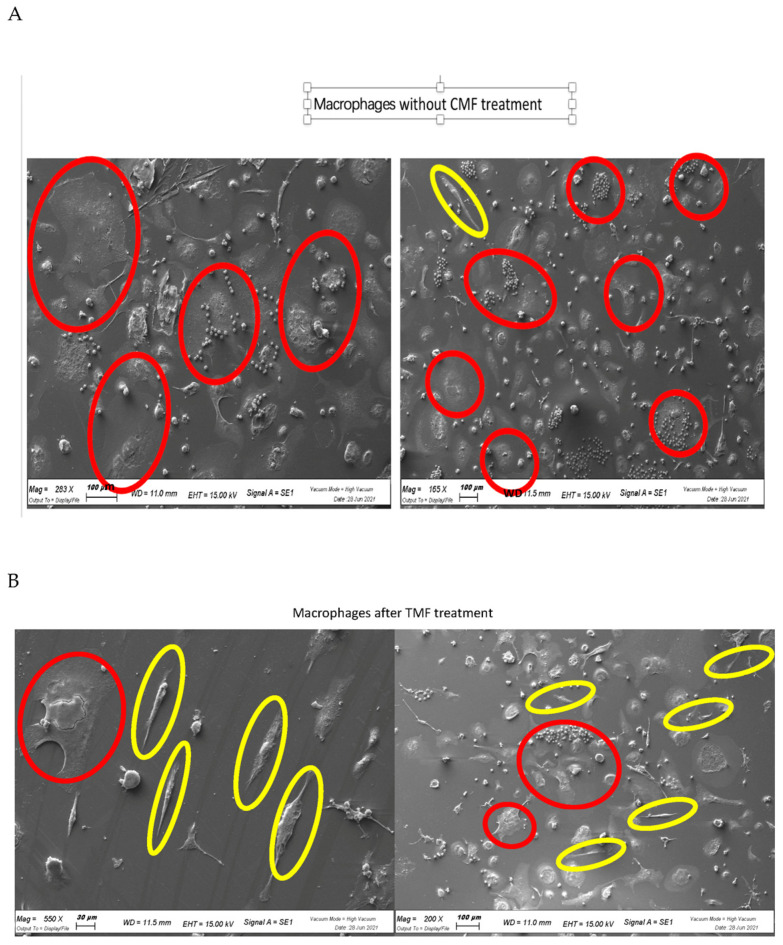
SEM analyses of the macrophages without the CMF treatment (**A**) acquires a M1 inflammatory rounded shape phenotype (red circles). Treatment with the CMF (**B**) induced the M2 anti-inflammatory phenotype characterized by a long fusiform shape (yellow circles).

This type of commitment from an anti-inflammatory phenotype M1 (red circles) characterized from a rounded shape to the anti-inflammatory phenotype M2 (elongated shape, yellow circles) were analyzed using electron microscopy. As reported in Figure 4, the treatment of the CMF increased the commitment into the M2 anti-inflammatory macrophages.

### 2.4. Anti-Inflammatory Markers

**Figure 5:**
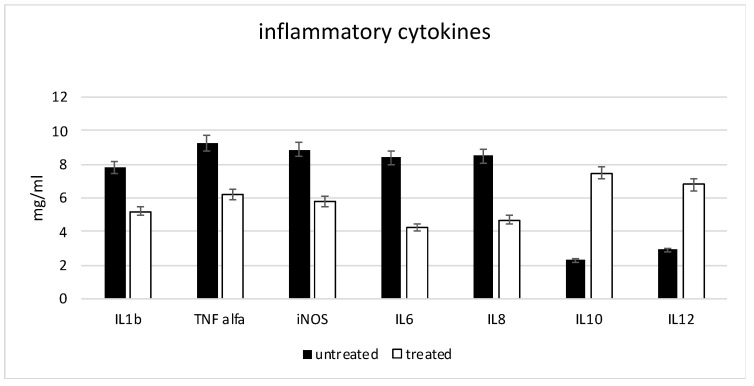
Gene expression related to the inflammatory markers. All the cells were cultured in the presence of an inflammatory condition; the black bars in the absence of the CMF, the white bars in the presence of the CMF.

The gene expression of inflammatory cytokines, such as IL1b, TNF-α, iNOS, IL6 and IL8, was detected on the macrophages cultures (Figure 5) in the presence of an inflammatory condition, confirming that, in such situations, the macrophages are in a M1 (inflammatory) phenotype. In contrast, interleukins with an anti-inflammatory activity such as IL10 and IL12 were less expressed. When the same protocols were performed in the presence of the CMF treatment (white bars), a strong reduction in inflammatory cytokines was observed and an increase in the anti-inflammatory cytokine expression was evident.

### 2.5. Extracellular Matrix

**Figure 6:**
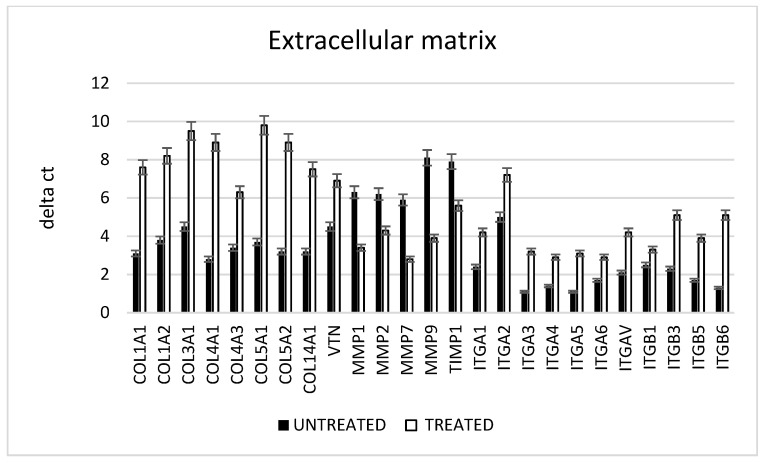
Gene expression related to collagen, remodeling enzyme and integrin expression. All the cells are cultured in the presence of an inflammatory condition; the black bars show normal conditions, the white bars show the presence of the CMF.

The ability of fibroblasts to act on the wound healing process was evaluated in vitro (Figure 6). The gene expression of the collagen fibers confirmed that, in the presence of an inflammation process, the fibroblasts did not produce a good extracellular matrix (ECM), such as collagen fibers type I, III, IV, V, XIV and vitronectin, as revealed by the low level of this gene expression (black bars). The presence of the metalloproteases (MMPs) such as MMP2 and 9, related to the destruction of the ECM, was otherwise high in the presence of inflammation. By contrast, the same inflammatory condition in the presence of the CMF induced an increased the ECM component, a decreased the enzymes directed to its digestion and increased the adhesion of proteins such as interleukins.

## 3. Discussion

In the field of non-invasive therapy to promote healing approved by the U.S. FDA, PEMF is a non-thermal treatment employing an active electromagnetic waveform in order to treat damaged tissues. The emitted electromagnetic waveforms are able to penetrate completely through any type of tissues as reported by Dr. Mattsson M.O. and Dr. Simkó M. [40,41,42,43,44,45,46]. This allows for the delivery of the therapy in a non-invasive way. The CMF based on biophysical energy was here used for the treatment of diabetic foot ulcers for accelerating their healing, evaluating the ROS production and their correlated biological event during an inflammatory event. When a chronic inflammation was induced, the principal factors associated with the failure of the healing of the diabetic foot ulcers were the high expression of the matrix metalloprotease and the ROS in the wound tissue [47,48,49,50,51,52]. The presence of the ROS induced damage and disrupted proteins, DNA and membrane phospholipids, and induced the diabetic wound impairment, whether in the prolonged presence of the pro-inflammatory (M1) macrophages phenotype or the related failure of their transition to the regenerative (M2) macrophage phenotype [53,54,55,56,57,58,59,60].

In this view, we first tested the CMF treatment for their safety, by means of the analysis in vitro following the ISO requirements. Different tests were performed as well as a mitochondrial activity assessment with MTT, an induction of mutagenesis with karyotyping and an AMES test in bacterial and hemolitic cells. All the results confirmed the total safety of the system. As second step, we searched for their ability to contrast the ROS production and to exert the anti-inflammatory activity. The ROS test confirmed that the CMF induced a reduction in these molecules, so we performed a real time PCR in order to evaluate the miRNA and cytokines involved in the inflammatory and anti-inflammatory events. The dysregulation of this macrophage plasticity was influenced by microRNAs (miRNAs) that are fundamental regulators of the transcriptome output. During wound healing, proinflammatory mediators such as lipopolysaccharide (LPS), TNF-α or IFN-γ  induced the polarization of the monocytes on the M1 macrophages phenotype. This led to an increased expression of the specific genes that favor extracellular matrix damage and the subsequent formation of ulcers.

In particular, miR-21, miR-155-5p, miR-204-5p, miR-451, miR-125b-5p, miR-181a-5p, miR-193b-3p, miR-125a-5p, Akt2, p110d, PTEN, TSC1, TSC2 and p85a play an essential role in the inflammatory immune response. In support for this, the expression of the genes related to the M2 phenotype (AKT1, p110a, p110b, p110g, TSC1 and RICTOR/mTORC2) was strongly reduced. However, the CMF treatment in an inflamed tissue strongly reduced the expression related to the M1 commitment (white bars, gene under the black bar) and strongly increased the genes related to the M2 phenotype (red bar, unlined genes). The previous results moreover confirmed that these miRNAs promoted the production of the reactive oxygen species (ROS) and the M1 polarization as well. Their expression was higher in an inflammatory environment, but in the presence of a PEMF, these values were significantly lower. In addition, the cells (M1 polarized macrophages and fibroblasts) exhibited an upregulation of pro-inflammatory markers such as TNFa, IL-6,IL-1b, iNos and IL-8 in the presence of inflammation, but a decrease in the presence of an EMF. The same trend occurred when we analyzed the extracellular matrix (ECM) component in the presence of the CMF in an inflammatory environment.

## 4. Materials and Methods

### 4.1. Patients Recruitment and Samples Collection

The ECAD-CLI (NCT03636867) is an investigator-driven, single-center, prospective, single-arm study enrolling patients who are admitted to the Diabetic Foot Unit of the Maria Cecilia Hospital (Cotignola, Italy) with a diagnosis of diabetes mellitus and critical limb ischemia (CLI) with DFU (consistent with the Rutherford classes 5 or 6). Forty patients were recruited at Maria Cecilia Hospital (Cotignola, Ravenna, Italy). The study was conducted following the ethical principles for medical research involving human subjects from the World Medical Association Declaration of Helsinki. The patients participated in the study after signing the consent form. The patients met the following inclusion criteria: they suffered from type 2 diabetes for at least 5 years, the presence of a distal neuropathic ulcer on the foot larger than 1 cm^2^ and appeared at least 6 weeks before, they had two palpable pulses at the ankle with a triphasic Doppler waveform and they were older than 18 years. The exclusion criteria were chronic renal failure in dialytic treatment, local ischemia with an ankle-brachial pressure index (ABPI) < 0.9; infection according to the Infective Diseases Societies of the Americas (IDSA) guidelines, active or chronic Charcot’s disease, HIV or any other systemic disease interfering with the immune system, steroid or cytostatic therapy, known or suspected cancer diagnosis and life expectancy of less than 1 year.

### 4.2. Cell Isolation and Characterization

The fibroblasts were isolated following our previous published protocol [61,62]. Briefly, the dermis was removed from biopsy, washed in a phosphate-buffered saline (PBS, EuroClone, Milano, Italy) added with 1% antibiotic–antimycotic (AA, Thermo Fisher Scientific, Waltham, MA, USA), minced and digested with 200 U/mL collagenase type II (Gibco, Thermo Fisher Scientific) in Hanks’ balanced salts solution (HBSS, Euroclone, Rome, Italy) at 37 °C for 16 h. The resulting cells were seeded at a density of 5 × 10^4^ cells/cm^2^ in Dulbecco’s modified eagle medium (DMEM, EuroClone, Rome, Italy) plus 10% fetal bovine serum (FBS, EuroClone, Rome, Italy). The isolation of the peripheral blood mononuclear cells (PBMCs) was carried out by a Ficoll–Paque gradient method. Briefly, the peripheral blood, freshly extracted from the patients, was carefully poured into a tube with the Ficoll at the blood/Ficoll 1:4 proportion, centrifuged at 591× *g* for 30 min at room temperature. The supernatant was discarded and the pellet containing the PBMCs was resuspended in 1 mL of PBS 1X for cell counting and viability tests. The cell cultures were maintained at 37 °C and 5% CO_2_ and the medium was changed twice a week. For mimicking the in vitro inflammatory conditions, the samples were treated for 24 h with 0.1 mg/mL^−1^ of the tumor necrosis factor-alpha (TNF-α, Celbio, Berlin, Germany). The TNF-α concentration used in the study was higher than in the physiologic conditions.

### 4.3. Treatment

The CMFs instrument, Next sx version (M.F.I. Medicina Fisica Integrata, Rome, Italy), is an electronic device that emits innovative pulsed multi-frequency electromagnetic fields between 1 and 250 microT variable in intensity, frequency, complex wave form and time stimulation.

The CMFs generator was provided with different programs that worked in relation to the configuration of the specific sector of the application.

Each program was composed of several different steps with different intensities (1–250 microT), frequencies (1–250 Hz), interval times (1–4 min each steps) and forms of the complex multi-frequency waves with harmonic enrichments.

Those four parameters, frequency, induction of intensity, wave form and time stimulation represented one of the steps of the machine program.

The machine program was normally formed using 6 to 10 steps.

All the details of the information regarding the machine programs were patent pending. Despite this, we noted that the parameters used in this work were a frequency from 1 to 112 Hz, an induction intensity from 1 to 195 μT, a time duration of the steps from 1 to 4 min each and impulsive waveforms with odd multiple harmonics.

The position of every step in the program followed the physiological priority of the biological project that we wanted to realize. For example, if we wanted to treat wound healing we had to consider several biological conditions, including the inflammation status, the excess of free radicals in the ROS, the fungal and bacterial conditions, the lack of vascularization and the lack of free energy through which the body could recreate the conditions for regeneration. The following Table (Table 3) explains the correlation between program and article related to the steps.

**Figure 7:**
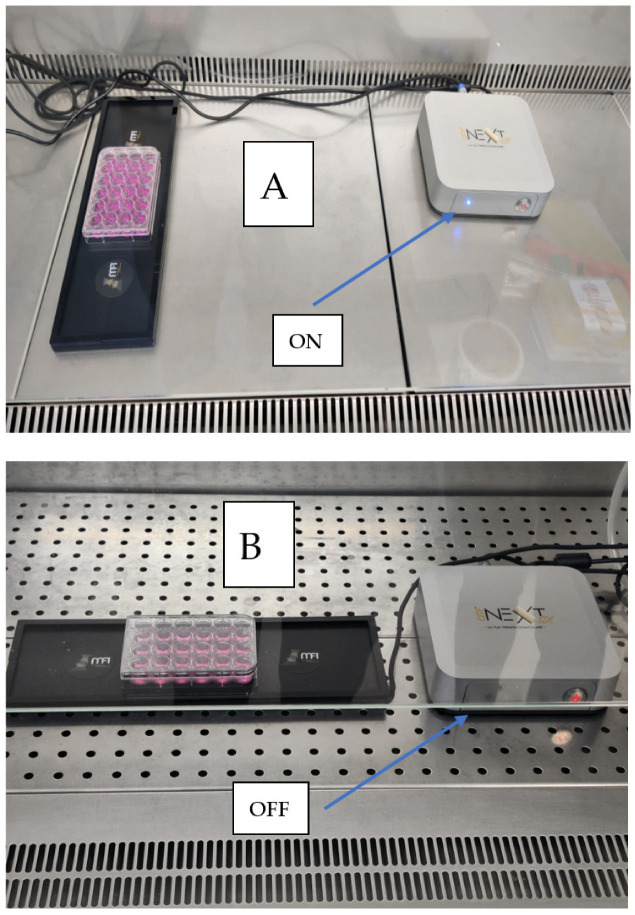
The treatment of the cell cultures. In detail, the cells cultured in 24-well plates were positioned corresponding to the experimental set-up, then exposed to the stimulation at room temperature (**A**) under biological flow. The control cultures were positioned onto the device in the same manner as the exposed cultures but without receiving stimulation (**B**).

In detail, the cells cultured in 24-well plates were positioned corresponding to the experimental set-up, then exposed to the stimulation at room temperature (Figure 7A) under the biological flow. The control cultures were positioned onto the device in the same manner as the exposed cultures but without receiving stimulation (Figure 7B).

### 4.4. In Vitro Cytotoxicity Test

The cytotoxicity of the treatment was evaluated in vitro using a mouse-derived established cell line of L929 fibroblasts (Cell bank Interlab Cell Line Collection, Genova, Italy) following the ISO 10993-5:2009 directions. The L929 cells were seeded at a density of 4 × 10^4^/well in 24-well plates for 24 h in the cDMEM medium. The cDMEM was created from Dulbecco’s modified eagle medium (DMEM) (Lonza S.r.l., Milano, Italy), supplemented with 10% fetal bovine serum (FBS) (Bidachem S.p.A., Milano, Italy) and 1% P/S. The cytotoxicity was assessed with the treatment for the cells. The negative control consisted of the fibroblasts seeded in in absence of the treatment; the blank was obtained by seeding the fibroblasts in the cDMEM with no test material added. Three samples were prepared for each group. The cytotoxicity produced for each different group was assessed with a 48 h cell exposure. After removing the test materials and the medium, 1 mL of a 0.5 mg mL-1 MTT solution was placed in each well. The MTT assay was then performed as previously explained. To determine the presence of viable cells, the MTT-based proliferation assay was performed according to the method of Denizot and Lang with minor modifications. Briefly, the tissue samples were incubated for 3 h at 37 °C in 1 mL of a 0.5 mg mL^−1^ MTT solution prepared in PBS. After the removal of the MTT solution by a pipette, 0.5 mL of 10% DMSO in isopropanol was added to extract the formazan in the samples for 30 min at 37 °C [16]. For each sample, the optical density (O.D.) values at 570 nm were recorded in duplicate on 200 μL aliquots deposited in the microwell plates using a multilabel plate reader (Victor 3, Perkin Elmer, Milano, Italy).

### 4.5. Hemolysis Assay

The hemolysis assay was performed following the standard practices set forth in the ASTM F756 for evaluating the blood compatibility of the cells after a treatment of the CMF and without treatment [29]. The blood of three healthy New Zealand rabbits was pooled and diluted in a phosphate buffer saline (PBS; Lonza S.r.l., Milano, Italy) to achieve a total hemoglobin concentration of 10 ± 1 mg/mL. One mL of this blood was added to 7 mL of the following PBS extracts: triplicate 2 g portions of Ti experimental or control implants in 10 mL PBS (test materials); triplicate 30 cm [2] portions of high-density polyethylene (HDPE) in 10 mL of the PBS (negative control); triplicate 10 mL portions of sterile water for injection (SWFI) (positive control). The extraction conditions were 50 °C for 72 h. Each sample was incubated for 3 h at 37 °C, then centrifuged for 15 min at 800× *g*. One mL of the resulting supernatant from all the samples was added to 1 mL of Drabkin’s reagent (Sigma-Aldrich, St. Louis, MO, USA) and incubated at room temperature for 15 min. The reaction product was quantified with a multilabel plate reader (Victor 3, Perkin Elmer, Milano, Italy) by measuring the optical density (OD) at 540 nm. The hemolysis index (HI) was then calculated using the mean OD for each group as follows.

HI(%) = OD (test material) − OD (negative control)/OD (positive control) − OD (negative control) × 100. For HI ≤ 2%, the sample was considered nonhemolytic; for HI > 2%, the sample was considered hemolytic.

### 4.6. Ames Test

The mutagenic potential of the CMF treatment was evaluated with the Ames test by using the Salmonella mutagenicity complete test kit (Moltox, Molecular toxicology Inc., Boone, NC, USA), as described in Ferroni et al. [30] Briefly, the Ti implants were extracted for (24 ± 2) h at (37 ± 1) °C, using a nutrient broth (blank) as the extraction vehicle. The same extraction conditions were set for the aluminum oxide ceramic rod (VITA In-Ceram Alumina CA-12, CE 0124, lot 15,320) (negative control) and the ICR 191 acridine (Moltox, 60–101) and sodium szide (Moltox, 60–103) (positive controls). Four different strains of Salmonella were incubated for 48 h at 37 °C with the different extracts, then the number of the revertant colonies per plate was counted. Three replicates were performed for each sample. If the number of the reverted colonies was equivalent to those observed with the blank and negative control, the sample was considered not mutagenic. If the number of the reverted colonies was equivalent to those observed with positive controls, the sample was considered mutagenic.

### 4.7. Karyotype Analysis

The fibroblast seeded onto the plastic dishes was exposed to the CMF treatment for 1 day. After 7 days, they were subjected to a karyotype analysis by capturing the metaphases using colchicine (Sigma-Aldrich) exposure for 6 h. The metaphases of the cells were stained by the Q-banding technique and karyotyped according to the international system for human cytogenetic nomenclature. Twenty-five metaphases were analyzed for the three expansions.

### 4.8. Scanning Electron Microscopy (SEM)

The samples were preserved in a 2.5% glutaraldehyde/0.1 M sodium cacodylate buffer overnight at 4 °C, treated with a 1% Osmio O4/0.1 M sodium cacodylate buffer and dehydrated using ethanol solutions of increasing concentrations. The samples were analyzed using SEM (Electronic Microscopy Service, Department of Biology, University of Padova, Padova, Italy) with a Tecnai G12 electron microscope (FEI Company, Hillsboro, OR, USA, acceleration voltage 100 kV). The image acquisition system consisted of a Tietz video camera (Tietz Video and Image Processing Systems GmbH, Gauting, Germany) and the TIA FEI imaging software 6 (FEI Company).

### 4.9. RNA Extraction and Real-Time PCR Array

The total RNA was extracted from the cells with the RNeasy Mini Kit (Qiagen, Hilden, Germany). Of each sample, 500 ng of the total RNA was reverse-transcribed with the RT2 First Strand Kit (Qiagen) in a SimpliAmp^TM^ Thermal Cycler (Applied Biosystems^TM^, Rome, Italy) Thermo Fisher Scientific, Berlin, Germany) following the manufacture indications. The resultant first-strand complementary DNA (cDNA) was stored at −20 °C until the next step. The human wound healing RT^2^ Profiler PCR Array (Qiagen) wwa performed in accordance with the manufacture protocol. Briefly, the cDNA samples were mixed with RT2 SYBR Green Mastermix (Qiagen) and then aliquoted into the wells of the RT^2^ Profiler PCR Array. The real-time PCR system (Applied Biosystems^TM^) was set up with the following thermal cycling conditions: denaturation at 95 °C for 10 min followed by 40 cycles of denaturation at 95 °C for 15 s and annealing and elongation at 60 °C for 1 min. A dissociation curve for each well was performed by running the following program: 95 °C for 1 min, 65 °C for 2 min and 65 °C to 95 °C at 2 °C/min. The relative expression was determined using the 2^−ΔΔCT^ method. The Ct values of the target genes were normalized to the geometric mean Ct values of the housekeeping gene (*ACTB*). The results were reported as a fold regulation of the target genes in the test group (treated with EMF) compared with the control group (no treated with EMF). The statistical significance was set at *p* < 0.05.

### 4.10. Reactive Oxygen Species (ROS) Measurements

The OxiSelect ROS Assay Kit (Cell Biolabs Inc., San Diego, CA, USA) is a cell-based assay for measuring the intracellular activity of hydroxyls, peroxyls and other ROSs employing the cell-permeable fluorogenic probe DCFHDA. It diffuses into the cells and is deacetylated by the cellular esterases into a non-fluorescent DCFH. In the presence of the ROS, the DCFH is rapidly oxidized to form highly fluorescent DCF. The fluorescence was read using a standard fluorometric plate reader.

### 4.11. Statistics

A one-way analysis of variance for data analyses was used. In addition, t-tests were used to ascertain the significant differences (*p* < 0.05). The repeatability was calculated as the standard deviation of the difference between the measurements. All the testing was performed in the SPSS 16.0 software (SPSS Inc., Chicago, IL, USA; license of the University of Ferrara, Ferrara, Italy).

## 5. Conclusions

**Figure 8:**
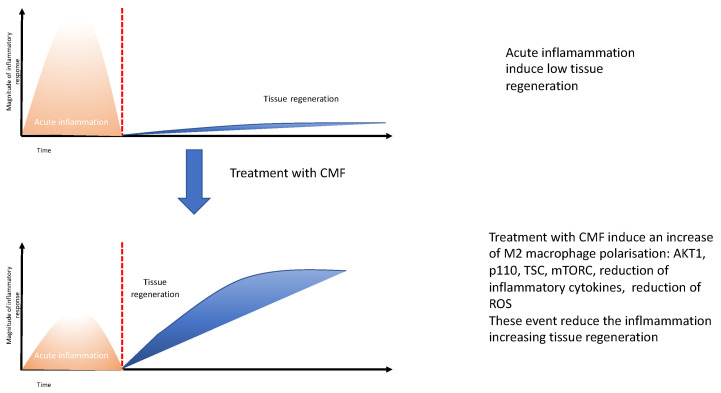
Conclusion of the potential effect of the CMF treatment on the wound healing process.

In light of these results, we conclude that the CMF induces a decrease in the ROS production in the presence of an inflammatory environment. Since it is well established that the ROS plays a crucial role in wound healing processes, including macrophages polarization, the killing of bacteria and the crosslinking of ECM, the reduction in the excessive amount of the ROS could improve tissue healing and regeneration as reported in Figure 8. These data confirm that EMF present in the CMF MD could strongly support tissue regeneration on chronic and difficult wounds, such diabetic foot.

## Figures and Tables

**Table 1:** Hemolitic test.

Sample	OD	Hemolysis Index	Results
Positive control	0.834 +/− 0.011	100%	Hemolytic
Negative control	0.0103 +/− 0.023	0%	Non Hemolitic
CMF treatment	0.0142 +/− 0.018	0.031%	Non Hemolitic
No treatment	0.0131 +/− 0.022	0.045%	Non Hemolitic

**Table 2:** AMES test.

	STDisc™ TA1535		STDisc™ TA1537		STDisc™ TA98		STDisc™ TA100	
Sample	Revertant Colonies	Mutagenic	Revertant Colonies	Mutagenic	Revertant Colonies	Mutagenic	Revertant Colonies	Mutagenic
Blank	4 ± 3	no	5 ± 3	no	4 ± 2	no	5 ± 2	no
Negative control	3 ± 2	no	3 ± 2	no	3± 2	no	2 ± 2	no
Positive control: ICR191	947 ± 85	yes	973 ± 66	yes	971 ± 79	yes	965 ± 69	yes
Positive control: Sodium Azide	853 ± 51	yes	876 ± 52	yes	893 ± 59	yes	879 ± 64	yes
CMF treatment	3 ± 2	no	2 ± 2	no	3 ± 2	no	3 ± 2	no
No treatment	3 ± 1	no	3 ± 2	no	2 ± 2	no	5 ± 2	no

**Table 3:** Steps and bibliography.

Mechanism of Action of Program: Wound Healing		
Program Step	Target	Bibliography
1	Anti-inflammatory	[63,64,65]
2	Normalization Intracellular cell communication	[66,67,68]
3	Antibacterial and anti fungal	[69,70,71,72,73]
4	ROS modulation	[74,75,76]
5	Normalization Intracellular cell communication	[66,67,68]
6	Vascularization and tissue engineering regeneration	[77,78,79]

## Data Availability

The data that support the findings of this study are available from the corresponding author upon reasonable request.
